# Patients with Inflammatory Bowel Disease Are Not at Increased Risk of COVID-19: A Large Multinational Cohort Study

**DOI:** 10.3390/jcm9113533

**Published:** 2020-10-31

**Authors:** Mariangela Allocca, María Chaparro, Haidee Aleman Gonzalez, Marta Maia Bosca-Watts, Carolina Palmela, Ferdinando D’Amico, Eirini Zacharopoulou, Uri Kopylov, Pierre Ellul, Giorgos Bamias, Vassilios Ntelis, Adi Lahat, Gerassimos J Mantzaris, Ioannis Papaconstantinou, Konstantinos Katsanos, Yulia Uspenskaya, Dimitrios Christodoulou, Shomron Ben Horin, Laurent Peyrin-Biroulet, Joanna Torres, Shaji Sebastian, Javier P Gisbert, Silvio Danese, Gionata Fiorino

**Affiliations:** 1 Humanitas Clinical and Research Center–IRCCS-, via Manzoni 56, 20089 Rozzano, Milan, Italy; eirinizachar@gmail.com (E.Z.); sdanese@hotmail.com (S.D.); 2 Department of Biomedical Sciences, Humanitas University, Via Rita Levi Montalcini 4, 20090 Pieve Emanuele, Milan, Italy; ferdinando.damico@humanitas.it (F.D.); gionataf@gmail.com (G.F.); 3 Gastroenterology Unit, Hospital Universitario de La Princesa, Instituto de Investigación Sanitaria Princesa (IIS-IP), Universidad Autónoma de Madrid, Centro de Investigación Biomédica en Red de Enfermedades Hepáticas y Digestivas (CIBEREHD), 28001 Madrid, Spain; mariachs2005@gmail.com (M.C.); javier.p.gisbert@gmail.com (J.P.G.); 4 IBD Unit, Hull University Hospitals NHS Trust, Hull HU1, UK; haidee.gonzalez@hey.nhs.uk (H.A.G.); shaji.sebastian@hey.nhs.uk (S.S.); 5 IBD Unit, Digestive Disease Department, University of Valencia, University Clinic Hospital of Valencia, 46010 València, Spain; maiabosca@yahoo.es; 6 Division of Gastroenterology, Hospital Beatriz Ângelo, 2620 Loures, Portugal; palmela.carolina@gmail.com (C.P.); joanatorres00@gmail.com (J.T.); 7 Department of Gastroenterology, Inserm U1256 NGERE, Nancy University Hospital, Lorraine University, 54500 Vandoeuvre-les-Nancy, France; peyrinbiroulet@gmail.com; 8 Department of Gastroenterology, Sheba Medical Center, Ramat Gan and Sackler School of Medicine, Tel Aviv University, Jaffa 59623, Israel; ukopylov@gmail.com (U.K.); zokadi@gmail.com (A.L.); shomron.benhorin@gmail.com (S.B.H.); 9 Division of Gastroenterology, Mater Dei Hospital, MT-34 Msida, Malta; ellul.pierre@gmail.com; 10 GI-Unit, 3rd Academic Department of Internal Medicine, Sotiria Hospital, Medical School, National and Kapodistrian University of Athens, 210 Athens, Greece; gbamias@gmail.com; 11 G.Gennimatas General Hospital, 210 Athens, Greece; basildelis@yahoo.gr; 12 Department of Gastroenterology, GHA Evaggelismos-Ophthalmiatreion Athinon-Polykliniki, 210 Athens, Greece; gjmantzaris@gmail.com; 13 2nd Academic Department of Surgery, Aretaieion Hospital, Medical School, National and Kapodistrian University of Athens, 210 Athens, Greece; johnpapacon@hotmail.com; 14 Division of Gastroenterology, University Hospital and University of Ioannina, 440 Ioannina, Greece; khkostas@hotmail.com (K.K.); dchristodoulou@gmail.com (D.C.); 15 Medical Center “Ne Bolit” LTT, Leninsky Prospect, 66 Moscow, Russia; jusp@mail.ru

**Keywords:** inflammatory bowel disease, immunosuppression, COVID-19, drugs

## Abstract

The impact of COVID-19 on inflammatory bowel disease (IBD) patients under pharmacological immunosuppression is still not clearly understood. We investigated the incidence of COVID-19 and the impact of immunosuppression and containment measures on the risk of SARS-CoV-2 infection in a large IBD cohort, from a multicenter cohort from 21st of February to 30th of June, 2020. Ninety-seven patients with IBD (43 UC, 53 CD, one unclassified IBD) and concomitant COVID-19 over a total of 23,879 patients with IBD were enrolled in the study. The cumulative incidence of SARS-CoV-2 infection in patients with IBD vs. the general population was 0.406% and 0.402% cases, respectively. Twenty-three patients (24%) were hospitalized, 21 (22%) had pneumonia, four (4%) were admitted to the Intensive Care Unit, and one patient died. Lethality in our cohort was 1% compared to 9% in the general population. At multivariable analysis, age > 65 years was associated with increased risk of pneumonia and hospitalization (OR 11.6, 95% CI 2.18–62.60; OR 5.1, 95% CI 1.10–23.86, respectively), treatment with corticosteroids increased the risk of hospitalization (OR 7.6, 95% CI 1.48–40.05), whereas monoclonal antibodies were associated with reduced risk of pneumonia and hospitalization (OR 0.1, 95% CI 0.04–0.52; OR 0.3, 95% CI 0.10–0.90, respectively). The risk of COVID-19 in patients with IBD is similar to the general population. National lockdown was effective in preventing infection in our cohort. Advanced age and treatment with corticosteroids impacted negatively on the outcome of COVID-19, whereas monoclonal antibodies did not seem to have a detrimental effect.

## 1. Introduction

The Coronavirus Disease 19 (COVID-19), a systemic infection caused by the new Severe Acute Respiratory Syndrome–Coronavirus 2 (SARS-CoV-2), has severely impacted on Health Systems and has led to a dramatic shrinkage of world economy. From the first cases reported in Wuhan, China, it has rapidly spread around the world, evolving into a pandemic. Currently, there are 43,038,798 confirmed cases worldwide, including 1,154,242 deaths (as for 25th October 2020) [1].

Inflammatory bowel disease (IBD), including Crohn’s disease (CD), ulcerative colitis (UC), and unclassified colitis, are chronic relapsing–remitting or continuously active idiopathic inflammatory bowel disorders, which affect millions of people worldwide and are characterized by an inappropriate, dysregulated immune response to as yet unidentified luminal antigens leading to over production of pro-inflammatory cytokines [2,3,4]. To control the intestinal inflammation, patients require frequent treatment with corticosteroids, immunosuppressants, and/or biologics at the cost of increased risk of infections, including influenza and pneumonia [5,6]. Despite concerns that patients with IBD treated with these agents could be more susceptible to COVID-19, so far there are no signals of an increased incidence or of more severe course of COVID-19 in this patient population [7,8]. Moreover, it has been hypothesized that some IBD medications could prevent the development of the lethal cytokine storm syndrome triggered by SARS-CoV-2 [9,10]. The impact of COVID-19 on patients with IBD is still poorly known, and one case of a patient with a severe UC treated with corticosteroids who died from pneumonia due to SARS-CoV-2 was reported [11]. Finally, steroid use has been associated with severe COVID-19 in patients with IBD [12,13].

The aim of this international multicenter study was to characterize COVID-19 in a large IBD cohort from 12 countries in Europe and Israel, in particular to evaluate its incidence from February 21st to June 30th, and the impact of immunosuppressive therapies and containment measures on the risk of SARS-CoV-2 infection.

## 2. Materials and Methods

This was a prospective, observational, international, multicenter cohort study. Twelve centers across Europe (Northwest Italy (Italy), Yorkshire (England), Lorraine (France), Madrid and Valencian Community (Spain), Lisbon (Portugal), Malta, Kastoria, Attica, Epirus (Greece), Moscow (Russian federation)), and Ramat Gan (Israel) participated in the study.

## 3. Study Population

All consecutive adult patients with IBD with COVID-19, from 21st February 2020 (first case in Italy) to 30th June, were included. The diagnosis of COVID-19 was either confirmed with a positive polymerase chain reaction (PCR) nasopharyngeal swab; or highly suspected in patients who did not undergo viral testing for limited availability of PCR testing to detect SARS-CoV-2, AND presented the typical symptoms (fever, dry cough, shortness of breath, anosmia, ageusia) AND had a history of a contact with an infected person OR had typical imaging features of COVID-19 pneumonia on a lung computed tomography [14]. For each patient, the following information was collected: country and region of residence, age, gender, IBD type, and activity (active disease was defined by a partial Mayo score > 2 for UC; and a Harvey–Bradshaw Index (HBI) > 4 for CD), current treatment, smoking, and comorbidities. Furthermore, exposure risk to SARS-CoV-2, including high-risk work (such as health workers, police officers, market clerks, etc.), contact with COVID-19 patients, no strict adherence to prevention measures (hand hygiene, mask wearing, social distancing, and staying at home, based on a questionnaire administered to the patient) and in- and out-patient visits (within 14 days from COVID infection) to evaluate the risk of being infected in hospitals and clinics were also explored.

## 4. Outcome Measures

The presence of gastrointestinal symptoms related to COVID-19, whether pneumonia occurred, whether the patient was hospitalized, and whether the patient developed complications (respiratory insufficiency, admission in intensive care units) and died of COVID-19 were recorded. The rate of COVID-19 was compared between patients infected during and outside (prior or after) the lockdown time period in each country. We arbitrarily decided to postpone the beginning and end of the lockdown to a week later, to avoid including patients infected prior to lockdown period but developed the disease during the lockdown due to the incubation period, or excluding patients who contacted the virus during the lockdown but developed the disease after the lockdown was waivered. The number of patients with IBD followed at each participating center was collected to calculate the cumulative incidence of COVID-19 and compare it with that in the general population for each country (https://qap.ecdc.europa.eu/public/extensions/COVID-19/COVID-19.html). The rate of lethality in the IBD cohort was also calculated and compared to that in the general population of each country (https://qap.ecdc.europa.eu/public/extensions/COVID-19/COVID-19.html).

## 5. Statistical Analyses

Descriptive statistics of the baseline data are presented as medians (interquartile range (IQR)) or as percentage when appropriate. Differences in qualitative parameters were tested using the χ^2^ test. The Wilcoxon test was used to compare differences in quantitative variables. A logistic regression analysis was performed to evaluate association and predictive role of patients’ characteristics and study outcomes (pneumonia, hospitalization, intensive care unit, death). Uni- and multivariable analyses were performed. All the variables resulted significantly associated with the study outcomes at univariable analysis were included in the multivariable analysis, with backward elimination of those variables which did not reach statistical significance in the model. A value of *p* < 0.05 was considered to be statistically significant.

## 6. Results

The overall cohort included 23,879 patients with IBD. Ninety-seven patients with IBD (43 UC, 53 CD, and 1 unclassified IBD) and concomitant COVID-19 had been registered by the end of the study (30th June). The diagnosis of SARS-CoV-2 infection was confirmed with a positive PCR nasopharyngeal swab in sixty-four patients (66%), while it was confirmed by clinical and radiological signs in 33 patients (34%), since those patients could not access to a nasopharyngeal swab. In Table 1, the demographic and clinical characteristics of these 97 SARS-CoV-2 positive patients with IBD and details on their treatments are reported.

## 7. The Impact of National Lockdowns on SARS-CoV-2 Infection

**Figure 1:**
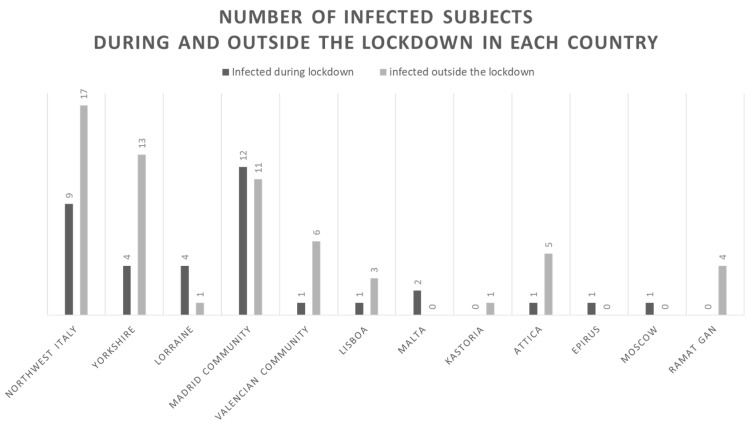
Graphical representation of the rate of infected subjects during and outside the lockdown for each country.

**Figure 2:**
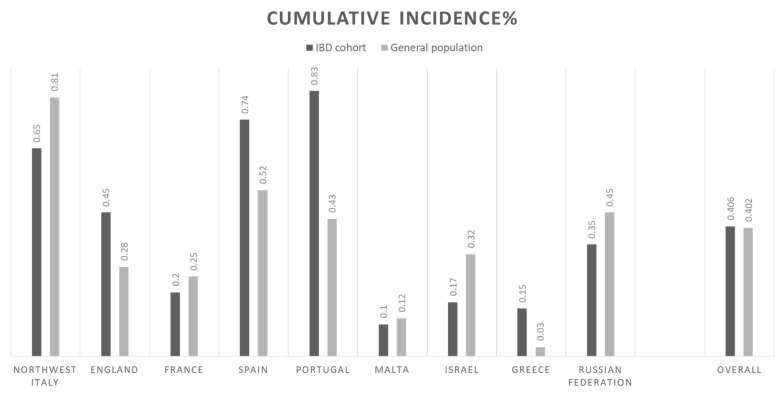
Graphical representation of the cumulative incidence of COVID-positive patients with IBD in our cohort and in the general population, overall and by country.

Figure 1 depicts the number of infected subjects during and outside the lockdown in each country. Overall, most of subjects were infected outside the lockdown, 61 (63%) compared to 36 (37%) during the lockdown. The overall cumulative incidence of COVID-19 infected patients with IBD in our cohort and in the general population were 0.406% and 0.402%, respectively. Figure 2 depicts the cumulative incidence overall and stratified by country. The lethality rate in our cohort was 1% and in the general population was 9%.

## 8. COVID-19 Outcomes

Twenty-one (22%) patients had pneumonia, 23 (24%) were hospitalized with a median (IQ range) stay of 14 days (9–21), four (4%) were admitted to the Intensive Care Unit (ICU) and one patient died. Three patients (two UC and one CD) were hospitalized for a severe disease flare when they were infected by the SARS-CoV-2. The patient who died was a 73-year-old patient from England suffering from Crohn’s ileo-colitis (L3 at Montreal classification) for 27 years and had a history of two prior surgeries. When he contracted the virus, his disease was in remission; despite that, he was not receiving any medications for CD, but he had relevant comorbidities, including chronic obstructive pulmonary disease, diabetes mellitus, cerebrovascular disease, and coronary artery disease. He was hospitalized and admitted to the ICU for pneumonia and died twenty days after the hospitalization.

At multivariable analysis, age > 65 years was positively associated with increased risk of pneumonia and hospitalization (OR 11.68, 95% CI 2.18–62.60; OR 5.13, 95% CI 1.10–23.86, respectively) (Table 2). Treatment with corticosteroids was also associated with an increased risk of hospitalization (OR 7.69, 95% CI 1.48–40.05) but treatment with monoclonal antibodies was associated with a significant reduced risk of pneumonia and hospitalization (OR 0.15, 95% CI 0.04–0.52; OR 0.31, 95% CI 0.10–0.90, respectively) (Table 2). No significant association was found between the defined variables with admission to ICU and death, due to the very small number of patients in these outcome groups (n = 4 and n = 1, respectively).

## 9. Special Situations

Two pregnant women with CD in remission (at 33 and 19 weeks of pregnancy, respectively) were infected. The first patient had diabetes mellitus and obesity and was receiving infliximab scheduled therapy. However, she was neither developing pneumonia nor was she hospitalized and finally delivered without complications. The second patient was receiving azathioprine and developed gastrointestinal symptoms but did not need hospitalization. She is continuing her pregnancy.

## 10. Discussion

The SARS-CoV-2 pandemic represents one of the major challenges in the recent time. The rapid spread throughout the world and the relatively high risk of complications and death have hugely impacted on human activities, and especially on healthcare systems.

The impact of COVID-19 on patients with immune-mediated diseases who require immunosuppressive and/or immunomodulators has been debated since the pandemic erupted. Up to now, some studies on local, national, and international cohorts have been conducted, generally showing that patients with IBD are not at higher risk of infection compared to the general population, but they also showed different results in terms of the impact of immunosuppressive therapies [7,13,15]. The preliminary results of the largest and still ongoing IBD–COVID-19 registry, SECURE-IBD (www.covidibd.org), reported that patients with IBD seem to have the same risk factors as the general population (advanced age and comorbidities). Regarding medications, while corticosteroids were associated with an increased risk of hospitalization, ventilation, ICU admittance, and also death, tumor necrosis factor antagonists were not [13]. We also found that the national lockdown was effective in containing and significantly decreasing the spread of infection in our cohort. As shown in Figure 1, the number of patients infected during the lockdown was lower than in the period soon before and after the lockdown phase. Moreover, no difference in the risk of infection was found based on the patient’s occupation, confirming that the general lockdown, together with the general recommendations on physical distance, washing hands, and using masks, were effective for all patients. Patients with IBD, especially if under immunosuppressive treatments, concerned for their safety, may take more stringent self-protective measures. Of note, medications with impact on immune system were not associated with increased risk of infection, except for steroids. This is consistent with the results of the SECURE registry [13]. We also found that monoclonal antibodies were associated with a significantly lower risk of hospitalization and COVID-19 pneumonia in our cohort. These findings suggest a potential protective or therapeutic role against the cytokine storm that has been described in COVID-19 patients and support the IOIBD recommendation about not discontinuing biological therapies in patients with IBD [16]. We also confirmed that the main risk factors for negative outcomes in COVID-19 patients were older age and comorbidities. The outcomes of patients with COVID-19 were consistent with previous data and not related to a high risk of mortality [13,15].

This study has some limitations. Firstly, a control group of non-IBD patients is lacking. However, we could estimate the power of our results based on the general population by country. Secondly, the number of patients is relatively small, and the rate of infections and deaths are considerably different across countries; however, this confirms that the SARS-CoV-2 infection rate in patients with IBD is not a major challenge compared to the general population. Moreover, we do not have data on the total number of patients with IBD found negative to the test and have no precise numbers on how many patients did the test elsewhere and could not be caught by our surveillance systems, although this possible selection bias is overcome by the fact that patients were actively contacted by the nurses at the beginning of the pandemic to give information about prevention and management, and by the fact almost all patients who were positive contacted the referral center for further instructions. Thirdly, we did not include all the hospitals and referral centers in the relevant region and countries; however, all the involved centers are local and national referral centers for patients with IBD. Finally, the lockdown was managed in a different way in each country, and this could be a potential limitation to be taken into account for the analysis of the period within and outside the lockdown.

In conclusion, patients with IBD are not at increased risk of SARS-CoV-2 infection, and COVID-19 negative outcomes. IBD-related medications, except for steroids, do not affect or even may improve the course of infection. General preventive measures, including the lockdown, may have been effective in containing the SARS-CoV-2 infection.

## Figures and Tables

**Table 1:** Characteristics of the patients with inflammatory bowel disease (IBD) and concomitant COVID-19 (N 97).

**Age at diagnosis of COVID-19**	42 (28.7–54.2)
**Age > 65 years**	9 (9.0)
**Males**	50 (52)
**Countries**	
Northwest Italy (Italy)	26 (27)
Yorkshire (England)	17 (18)
Lorraine (France)	5 (5.0)
Madrid (Spain)	23 (24)
Valencia (Spain)	7 (7.0)
Lisboa (Portugal)	5 (5.0)
Malta (Malta)	2 (2.0)
Ramat Gan (Israel)	4 (4.0)
Kastoria (Greece)	1 (1.0)
Athens (Greece)	5 (5.0)
Epirus (Greece)	1 (1.0)
Moscow (Russian federation)	1 (1.0)
**IBD diagnosis**	
Ulcerative colitis	43 (44)
Crohn’s disease	53 (55)
Unclassified IBD	1 (1.0)
Clinical activity *	27 (28)
**Medications**	
Corticosteroids	8 (8.0)
Any immunosuppressants	24 (25)
Azathioprine	16 (67)
Methotrexate	7 (29)
Tacrolimus	1 (4.0)
Monoclonal Antibodies	51 (53)
Tumor necrosis factor inhibitor	38 (74)
Interleukin-12/23 blocker	10 (20)
Vedolizumab	3 (6.0)
Janus Kinase Inhibitor	1 (1.0)
Filgotinib	1 (1.0)
**Combo therapy**	18 (19)
Azathioprine + Tumor necrosis factor inhibitor	8 (44)
Methotrexate + Tumor necrosis factor inhibitor	3 (17)
Methotrexate + Interleukin-12/23 blocker	3 (17)
Corticosteroids + Tacrolimus + Tumor necrosis factor inhibitor	1 (5.0)
Corticosteroids + Tumor necrosis factor inhibitor	2 (11)
Corticosteroids + Tumor necrosis factor inhibitor	1 (5.0)
**Clinical trial participants**	4 (4.0)
**Exposure risk**	
High-risk work **	18 (22)
Working contact	42 (48)
Contact with confirmed or suspected COVID patients	44 (56)
No strict adherence to prevention measures	27 (28)
In- and out-patient visits (within 14 days from COVID infection)	22 (23)
**Smoking**	8 (8.0)
**Comorbidities**	33 (34)
Hypertension	13 (13)
Chronic lung diseases	3 (3.0)
Diabetes mellitus	5 (5.0)
Obesity	5 (5.0)
Connective tissue diseases	6 (6.0)
Malignancy ^§^	2 (2.0)
Chronic liver disease	4 (4.0)
Stroke	1 (1.0)
Myocardial ischemia	2 (2.0)
Renal transplantation	1 (1.0)
Other	3 (3.0)
**Pregnancy**	2 (2.0)
**Positive rhino-pharyngeal swabs**	64 (66)
**COVID-related symptoms**	87 (90)
Gastro-intestinal symptoms	30 (31)
**COVID-related pneumonia**	21 (22)
**Discontinuation of IBD therapy**	46 (47)
**Hospitalization**	23 (24)
Intensive Care Unit	4 (4.0)
**Death**	1 (1.0)

IBD: inflammatory bowel disease; data are presented as medians (interquartile range) or percentages when appropriate; * disease activity was defined as partial Mayo score > 2 in UC and HBI > 4 in CD; ** 15 health workers, 1 police officer, 1 truck driver, 1 market clerk; ^§^ 1 renal carcinoma, 1 colon cancer.

**Table 2:** Influence of baseline parameters on the risk of pneumonia and hospitalization.

	Risk of Pneumonia				Risk of Hospitalization			
	Univariate Analysis		Multivariate Analysis		Univariate Analysis		Multivariate Analysis	
Parameters	OR (95% CI)	*p*	OR (95% CI)	*p*	OR (95% CI)	*p*	OR (95% CI)	*p*
**Age > 65 years**	9.60 (2.15–42.74)	0.003	6.76 (1.10–41.46)	**0.038**	4.86 (1.18–19.97)	0.028	5.13 (1.10–23.86)	**0.036**
**Any comorbidity**	4.46 (1.61–12.37)	0.004	-	-	2.75 (1.05–7.20)	0.039	-	-
**Charlson’s Comorbidity Index** **(per unit increase)**	1.47 (1.06–2.04)	0.02	-	-	1.40 (1.021.91)	0.03	-	-
**Monoclonal Antibodies**	0.17 (0.05–0.53)	0.002	0.15 (0.04–0.54)	**0.003**	0.32 (0.12–0.86)	0.024	0.31 (0.10–0.90)	**0.031**
**Diagnosis of UC ^§^**	3.17 (1.14–8.79)	0.02	-	-	1.90 (0.74–4.91)	0.18	-	-
**Male gender**	2.85 (1.00–8.16)	0.05	-	-	2.08 (0.79–5.52)	0.13	-	-
**Corticosteroids**	2.33 (0.51–0.69)	0.27	-	-	6.57 (1.43–30.11)	0.015	7.69 (1.48–0.05)	**0.015**
**Immunomodulators**	0.92 (0.29–2.85)	0.88	-	-	1.09 (0.37–3.20)	0.86	-	-
**Combination therapy**	0.38 (0.08–1.84)	0.23	-	-	0.90 (0.26–3.07)	0.87	-	-
**Smoking habit**	0,42 (0.04–3.55)	0.42	-	-	0.32 (0.03–2.74)	0.30	-	-

^§^ UC, ulcerative colitis.
